# Differential expression of the TFIIIB subunits Brf1 and Brf2 in cancer cells

**DOI:** 10.1186/1471-2199-9-74

**Published:** 2008-08-12

**Authors:** Stephanie Cabarcas, Joby Jacob, Ingrid Veras, Laura Schramm

**Affiliations:** 1 Department of Biological Sciences, St. John's University, 8000 Utopia Parkway, Queens, NY 11439, USA

## Abstract

**Background:**

RNA polymerase (pol) III transcription is specifically elevated in a variety of cancers and is a target of regulation by a variety of tumor suppressors and oncogenes. Accurate initiation by RNA pol III is dependent on TFIIIB. In higher eukaryotes, two forms of TFIIIB have been characterized. TFIIIB required for proper initiation from gene internal RNA pol III promoters is comprised of TBP, Bdp1, and Brf1. Proper initiation from gene external RNA pol III promoters requires TBP, Bdp1, and Brf2. We hypothesized that deregulation of RNA polymerase III transcription in cancer may be a consequence of altered TFIIIB expression

**Results:**

Here, we report: (1) the TFIIIB subunits Brf1 and Brf2 are differentially expressed in a variety of cancer cell lines: (2) the Brf1 and Brf2 promoters differ in activity in cancer cell lines, and (3) VAI transcription is universally elevated, as compared to U6, in breast, prostate and cervical cancer cells.

**Conclusion:**

Deregulation of TFIIIB-mediated transcription may be an important step in tumor development. We demonstrate that Brf1 and Brf2 mRNA are differentially expressed in a variety of cancer cells and that the Brf2 promoter is more active than the Brf1 promoter in all cell lines tested. We also demonstrate, that Brf1-dependent VAI transcription was significantly higher than the Brf2-dependent U6 snRNA transcription in all cancer cell lines tested. The data presented suggest that Brf2 protein expression levels correlate with U6 promoter activity in the breast, cervical and prostate cell lines tested. Interestingly, the Brf1 protein levels did not vary considerably in HeLa, MCF-7 and DU-145 cells, yet Brf1 mRNA expression varied considerably in breast, prostate and cervical cancer cell lines tested. Thus, Brf1 promoter activity and Brf1 protein expression levels did not correlate well with Brf1-dependent transcription levels. Taken together, we reason that deregulation of Brf1 and Brf2 expression could be a key mechanism responsible for the observed deregulation of RNA pol III transcription in cancer cells.

## Background

RNA polymerase III (RNA pol III) is the largest of the eukaryotic DNA dependent RNA polymerases and transcribes many of the genes involved in mRNA processing (U6 snRNA) and protein translation (tRNA), thereby regulating the growth rate of a cell [1]. RNA pol III activity has been demonstrated to be deregulated in a variety of cancers, irrespective of tissue type (reviewed in [2-4]). Like all eukaryotic RNA polymerases, RNA pol III cannot recognize target promoter elements directly. Proper initiation by RNA pol III requires the transcription factor TFIIIB [1,5]. In higher eukaryotes, two forms of TFIIIB have been identified thus far [6-8]. The form of TFIIIB required for proper initiation from gene internal RNA pol III promoters is comprised of TBP, Bdp1, and Brf1 [9,10]. Proper initiation from gene external RNA pol III promoters requires TBP, Bdp1, and Brf2 [6-8].

TFIIIB is a molecular target of regulation by a wide variety of tumor suppressors, including p53 [11-15], ARF [16], PTEN [17], RB [18,19], and the RB-related pocket proteins [20], as well as the oncogene c-myc [18,21] and the mitogen-activated protein kinase ERK [22]. RNA pol III and TFIIIB activity have also been demonstrated to be negatively regulated by other proteins, such as Maf1 [23-26]. Maf1 is a common component of at least three signaling pathways leading to repression of RNA pol III transcription: the DNA damage signaling pathway, the secretory defect signaling pathway, and the target of rapamycin (TOR) signaling pathway (reviewed in [27,28]). More recently, RNA pol III transcription has also been demonstrated to be negatively regulated by the chemopreventative agent EGCG [29].

Taken together, these data suggest that deregulation of TFIIIB-mediated transcription may be an important step in tumor development. Thus, we hypothesized that the observed specific elevation of RNA pol III transcription products in cancer (reviewed in [2,4,30]) may be a result of alterations in expression of the RNA pol III initiation factor TFIIIB. Here, we report that the TFIIIB subunits Brf1 and Brf2 are differentially expressed in a variety of cancer cell lines. We further demonstrate that the Brf1 and Brf2 promoters differ in activity in cancer cell lines, and VAI transcription is universally elevated, as compared to U6 snRNA transcription, in breast, prostate and cancer cell lines.

## Results and Discussion

### The TFIIIB subunits Brf1 and Brf2 are differentially expressed in cancer cell lines

**Figure 1:**
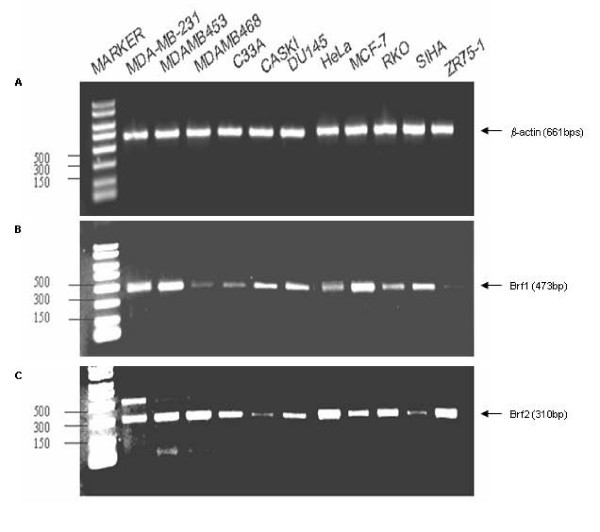
**The TFIIIB subunits Brf1 and Brf2 are differentially expressed in cancer cell lines**. Total RNA was isolated from asynchronous growing Caski, HeLa, RKO, SIHA, Du 145, ZR75-1, MCF-7, MDA-MB-453, MDA-MB-231, and MDA-MB-468 cells. After first strand cDNA synthesis, diluted cDNA was used in PCR using the primers depicted in Table 1. The mRNA expression of (A) β-actin, (B) Brf1, and (C) Brf2 are shown. The expected sizes of the PCR products of the different TFIIIB subunits are depicted.

Accurate initiation of transcription by RNA pol III requires TFIIIB. Two of the TFIIIB subunits, Brf1 and Brf2, are structurally related and are required for accurate transcription by RNA pol III [1,7]. Brf1 is required for transcription from gene internal promoters (tRNA), whereas Brf2 is required for transcription from gene external RNA pol III promoters (U6 snRNA). Hence, we sought to determine if Brf1 and Brf2 expression is elevated in a variety of cancer cell lines, contributing to the previously observed increase of RNA pol III transcripts in cancer. We obtained a variety of cervical, colorectal, breast, and prostate cancer cell lines from the ATCC, isolated total RNA from asynchronous cells, and by RT-PCR determined the expression levels of Brf1 and Brf2 using gene specific primers (Table 1). Primers for β-actin (Table 1) were used as a control, Figure 1A. Strikingly, the TFIIIB subunits Brf1 and Brf2 are differentially expressed in breast, cervical and prostate cancer cell lines (Figure 1B and 1C). We then speculated that the observed differences in Brf1 and Brf2 expression is a consequence of alterations in mRNA stability or may be a result of differential activity of the Brf1 and Brf2 promoters.

**Table 1:** Primers used for semiquantitative RT-PCR analyses

**Gene**	**Forward Primer**	**Reverse Primer**
**β-actin**	5'-tagcggggttcacccacactgtgccccatcta-3'	5'-ctagaagcatttgcggtggaccgatggaggg-3'
**Brf1**	5'ggcattgatgacctggagat-3'	5'accagaggcctcaacctttt-3'
**Brf2**	5'cagaagtggagacccgagag-3'	5' cagggagggttagggacact-3'

### The Brf1 and Brf2 promoters differ in activity in breast, cervical and prostate cancer cell lines

**Figure 2:**
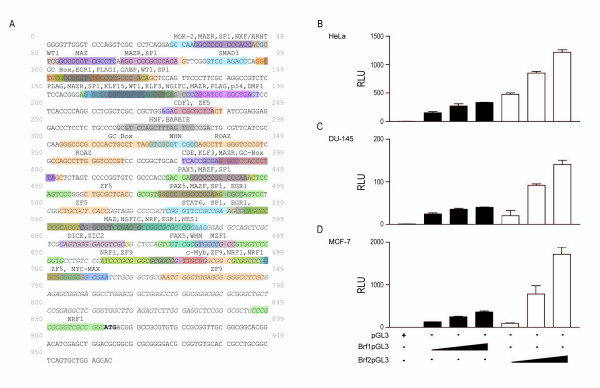
**The Brf1 and Brf2 promoters differ in activity in breast, cervical and prostate cancer cell lines**. (A) Schematic representation of the Brf1 promoter identified. The empty pGL3 vector (300 ng), as well as increasing concentrations of Brf1-pGL3 (100 ng, 200 ng, 300 ng) and Brf2-pGL3 (100 ng, 200 ng, 300 ng) were transiently transfected into asynchronous (A) HeLa, (B) DU145, and (C) MCF-7 cells. All luciferase assay results are expressed as relative light units (RLU): the average of the Photinus pyralis firefly activity observed divided by the average of the activity recorded from the Renilla luciferase vector. Experiments were done in triplicate, repeated three times, and representative experiments are depicted.

To determine if the observed differences in Brf1 and Brf2 expression is a result of differences in transcription initiation, we set out to directly compare the activities of the Brf1 and Brf2 promoters. We have previously cloned the human Brf2 promoter [29], and using a similar strategy we cloned the human Brf1 promoter. Using Gene2Promoter software of the Genomatix Suite, we identified a putative promoter for Brf1, Figure 2A. We subsequently amplified this putative promoter by PCR and sub-cloned it into a promoterless pGL3-Basic vector, generating Brf1-pGL3. As expected, Brf1-pGL3 was able to drive luciferase expression in a DNA concentration-dependent manner in all three cell lines tested: HeLa, DU145 and MCF-7 (Figure 2). These cell lines were selected since they all differentially expressed Brf1 and Brf2. HeLa cells expressed higher levels of Brf2 than Brf1, Figure 1. MCF-7 cells expressed higher levels of Brf1, as compared to Brf2, Figure 1. The DU145 cell line expressed only slightly higher levels of Brf1, as compared to Brf2 (Figure 1). Strikingly, in all three cell lines tested, the Brf2 promoter activity was significantly higher than the Brf1 promoter, Figure 2B–D, irrespective of Brf1 and Brf2 mRNA expression levels previously determined (Figure 1).

We cannot currently rule out the possibility that the observed lower Brf1 promoter activity is a consequence of enhancer sequences missing in the Brf1pGL3 construct used in these studies. However, analysis of the Brf1 promoter reveals several cis-acting elements which may negatively regulate expression of Brf1. The Brf1 promoter contains several binding sites for Krüppel-like zinc finger transcription factors similar to Sp1, classified into structural subfamilies derived from variant sequences within their N-terminal domains and termed KLF (Krüppel-like factor) [31]. The Brf1 promoter has three binding sites for the tumor suppressor ZF9, also known as KLF6 [32], which could negatively regulate expression from the Brf1 promoter. Also, the Brf1 promoter contains multiple KLF3 binding sites, previously identified as a CtBp-mediated repressor protein [31]. In addition, two ZF5 cis-regulatory elements also are found within the Brf1 promoter. ZF5 is a ubiquitous Kruppel-like zinc protein originally identified as a negative regulator of the c-myc promoter [33]. Taken together, these observations suggest that negative regulation of the Brf1 promoter may account for the consistently lower levels of Brf1 promoter activity as compared to Brf2 in all cell lines tested (Figure 2).

The Brf1 promoter also contains binding sites for transcription factors which could positively regulate Brf1 expression. For example, the Brf1 promoter contains a Myc/Max binding site. Sansom et al, demonstrated using a double mutant for Myc that Brf1 was no longer transcriptionally upregulated in the absence of functional Myc [34]. However, it is unclear if regulation of Brf1 expression by Myc is a direct or indirect effect [34]. The Brf1 promoter also contains several binding sites for nuclear respiratory factor 1(NRF1), which has been implicated in the upregulation of genes involved in fundamental cellular activities [35] and mitochondrial respiratory function [36]. Additionally, the Brf1 promoter contains a binding site for Zic2, a transcriptional activator that plays a role in mammalian forebrain development [37]. The Brf1 promoter also contains an immediate early growth response (EGR1) binding site. EGR1 is required for programmed cell death or apoptosis in both normal and tumor cells, but has also been determined to stimulate the differentiation of several cell types [38]. Hence, positive and negative regulation of Brf1 expression may be a key event in regulating the growth rate of a cell.

### VAI transcription is universally higher than U6 snRNA transcription, in breast, prostate and cancer cell lines

**Figure 3:**
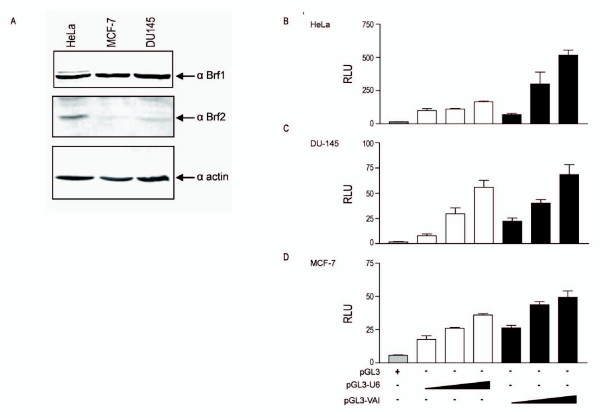
**VAI transcription is higher than U6 snRNA transcription in HeLa, DU145, and MCF-7 cells**. The empty pGL3 vector (300 ng), as well as increasing concentrations of pGL3-U6 (100 ng, 200 ng, 300 ng) and pGL3-VAI (100 ng, 200 ng, 300 ng) were transiently transfected into asynchronous (A) HeLa, (B) DU145, and (C) MCF-7 cells. All luciferase assay results are expressed as relative light units (RLU): the average of the Photinus pyralis firefly activity observed divided by the average of the activity recorded from the Renilla luciferase vector. Experiments were done in triplicate, repeated three times, and representative experiments are depicted. (D) Western blot analysis of Brf1 and Brf2 protein levels in HeLa, MCF-7 and DU145 cells. 25 μg of nuclear extract was immunoblotted with anti-Brf1 (CS1043) and anti-Brf2 (CS1228) antibodies. As loading control, membrane was also immunoblotted with an anti-actin antibody, bottom panel. Arrows depict migration of actin, Brf1 and Brf2.

The interesting observations that the Brf2 promoter is more active than the Brf1 promoter in all cell lines tested and that Brf1 and Brf2 mRNA are differentially expressed in cervical, breast and prostate cancer cell lines led us to speculate what effects these observations have on protein expression levels of Brf1 and Brf2. Thus, we determined the endogenous protein levels of Brf1 and Brf2 in HeLa, MCF-7 and DU145 cells (Figure 3A). The Brf1 protein levels did not vary considerably in the breast, cervical and prostate lines tested (Figure 3A). However, the Brf2 protein expression levels differed significantly between the cervical, breast and prostate cancer cell lines tested, as compared to β-actin (Figure 3A).

We then speculated what effect Brf1 and Brf2 protein concentrations had on VAI and U6 snRNA transcription levels in breast, prostate and cervical cancer cell lines. To address this issue we transiently transfected asynchronous HeLa, MCF-7, and DU145 cells with increasing concentrations of the U6 snRNA (pGL3-U6) and the VAI (pGL3-VAI) promoters [26]. In all three cell lines tested, VAI transcription levels were higher than U6 snRNA, Figure 3B–D. This result was surprising as we have previously observed that Brf1 and Brf2 are differentially expressed (Figure 1) and the U6 specific TFIIIB subunit Brf2 promoter activity was significantly higher than Brf1 promoter activity in all three cell lines tested (Figure 2). Although the Brf1 protein levels did not vary much in HeLa, MCF-7 and DU-145 cells, VAI transcription levels in these cancer cell lines varied considerably. VAI transcription was approximately five fold higher in HeLa cells (Figure 3B), as compared to DU-145 (Figure 3C) and MCF-7 cells (Figure 3D). One possible reason that the observed VAI transcription levels did not correlate well with Brf1 protein levels is that cancer cells may already express Brf1 levels far above limiting concentrations for RNA pol III transcription. Interestingly, there is a correlation between Brf2 protein levels (Figure 3A) and the activity of the U6 promoter in HeLa (Figure 3B), DU-145 (Figure 3C) and MCF-7 (Figure 3D) cells.

We speculate the differences in Brf1 and Brf2 mRNA expression and promoter activities, as well as the observed differences in TFIIIB-mediated RNA pol III transcription is a consequence of differences in Brf1 and Brf2 mRNA stability. Differences in Brf1 and Brf2 mRNA stability may, in part, account for the differences in the endogenous protein levels of Brf1 and Brf2 observed in Figure 3A, as compared to mRNA expression levels in Figure 1B–C.

Modulation of mRNA stability provides a rapid mechanism for regulating protein levels, independent of transcription initiation. A major mechanism controlling the rate of mRNA turnover is the regulation of destabilizing cis-regulatory elements within a given transcript [39]. mRNA destabilizing cis-regulatory elements have been localized as coding region determinant (CRD) sequences [40-43], or as the more common AU-rich elements (ARE) in the 3'UTR [44] of a transcript. These AU-rich elements are heterogenous in terms of length, AU-content and 3'UTR location. However, AREs may be classified based on sequence criteria [42,45,46]. Class I AREs contain multiple canonical AUUUA sites within the 3' UTR and Class II contains multiple AUUUA sites which may overlap resulting in an AUUUAUUUA sequence. The Class III AREs have U-rich regions in the 3'UTRs, without a canonical AUUUA site.

Using the ElDorado software of the Genomatix suite we identified 3'UTRs for Brf1 and Brf2 (data not shown). The 3'UTRs of Brf1 and Brf2 were AU-rich, 25% and 52% respectively, but do not appear to contain classical Class I or Class II AU-rich elements. However, it remains to be experimentally determined if the significant AU-content of the 3'UTR of Brf2 plays a role in regulating Brf2 mRNA turnover.

**Figure 4:**
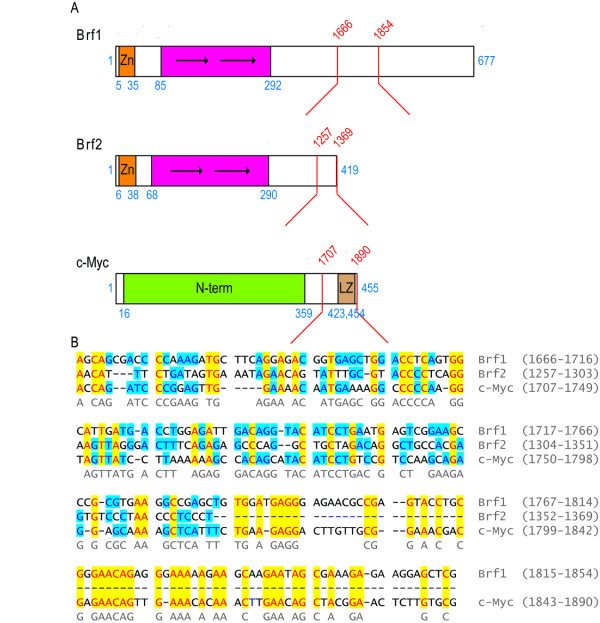
**Brf1 and Brf2 have a high sequence similarity to the codon determinant region of c-myc**. (A) Schematic representation of Brf1, Brf2 and c-myc. Structural features are indicated. Location of nucleotide sequences aligned (red text) in lower panel are indicated by red brackets. (B) Sequence alignment of the codon determinant region of c-myc, Brf1 and Brf2. Identical amino acids in all three sequences are depicted in yellow. Sequences identical in two of the three sequences are highlighted in blue. Consensus sequence is indicated.

We cannot currently rule out the possibility that Brf1 and Brf2 mRNA stability is regulated by coding region determinant elements as previously described for c-fos [41], c-myc [40,43], and MnSOD [47]. Using Align X (Invitrogen), we aligned the coding sequences of Brf1 (ntds 1666–1858) and Brf2 (ntds 1257–1456) with a c-myc nucleotide sequence corresponding to a 60 amino acid region (372–412) previously defined as a coding region determinant [40,43] (Figure 4). Strikingly, within the coding regions for the c-terminus of Brf1 and Brf2, there was a high degree of sequence identity with the coding determinant region of c-myc: Brf1 was 50.5% identical and Brf2 50.1%. We will experimentally determine whether these regions indeed influence Brf1 and Brf2 mRNA stability, ultimately providing an additional mechanism for regulation of basal RNA pol III transcription factors.

## Conclusion

We have demonstrated that the TFIIIB subunits Brf1 and Brf2 are differentially expressed at the mRNA level in a variety of cancer cells (Figure 1). We also show that the Brf2 promoter is more active than the Brf1 promoter in the breast, prostate and cervical cell lines tested (Figure 2B–D). Brf1-dependent VAI transcription was significantly higher than the Brf2-dependent U6 snRNA transcription in HeLa, DU-145 and MCF-7 cell lines (Figure 3B–D). Surprisingly, the Brf1 protein levels did not vary considerably in HeLa, MCF-7 and DU-145 cells (Figure 3A), yet Brf1 mRNA expression varied considerably in the cancer cell lines tested (Figure 1B). Thus, Brf1 promoter activity (Figure 2B–D) and Brf1 protein expression (Figure 3A) levels did not correlate well with Brf1-dependent transcription levels (Figure 3B–D).

Interestingly, we have observed that Brf2 protein levels (Figure 3A) may correlate with U6 snRNA transcription rates in the breast, cervical and prostate cancer cell lines tested (Figure 3B–D). Hence, we speculate that since Brf2 has been shown to be amplified in breast cancers and has been proposed to be a candidate oncogene [48,49], strict regulation of Brf2 expression could be critical in preventing the oncogenic phenotype. Also, Brf1 expression has been demonstrated to be induced in cervical cells infected with papilloma virus [50] and cardiomyocytes undergoing hypertrophy [51,52]. In addition, Brf1 has been shown to be a direct target of activation by c-myc and ERK [21,22], both known to play a key role in cancer pathogenesis. Taken together, we reason that deregulation of Brf1 and Brf2 expression could be a key mechanism responsible for the observed deregulation of RNA pol III transcription in cancer cells.

## Methods

### Cell Lines and Culture

C33A, Caski, HeLa, RKO, SiHa, DU145, ZR75-1, MCF-7, MDA-MB-453, MDA-MB-231, and MDA-MB-468 cells were obtained from the American Type Culture Collection (Rockville, MD). Cells were cultured in DMEM or RPMI supplemented with FCS (5% v/v), nonessential amino acids (100 mM), L-glutamine (5 mM), streptomycin (100 μg/ml), and penicillin (100 units/ml; all from BioWhittaker, Walkersville, MD). Cells were grown at 37°C in a humidified atmosphere of 95% air and 5% CO_2_.

### Total RNA Isolation

Total RNA was extracted from the subconfluent 100 mm dishes of the cancer cell lines indicated using TRIzol Reagent (Life Technologies, Inc.), according to the manufacturer's protocol. The RNA was DNase (Ambion) treated and ethanol precipitated prior to cDNA synthesis. Isolated RNA was electrophoresed through 1.0% agarose-formaldehyde gels to verify the quality of the RNA, and RNA concentrations were determined from absorbance measurements at 260 and 280 nm.

### Semiquantitative RT-PCR Analysis

Aliquots of total cellular RNA (1.0 μg) were subjected to first-strand cDNA synthesis using Superscript II reverse transcriptase (Life Technologies, Inc.), and the cDNA was diluted five times with water and 1μl of the diluted cDNA was used for each PCR reaction. PCR amplifications were performed using a Techgene TC312 DNA thermal cycler. The PCR primer sets used in this study are shown in Table 1. The PCR reaction conditions were individually optimized for each gene product tested in this study. For each gene product, the cycle number was adjusted so that the reactions fell within the linear range of product amplification. The β-actin gene was used as a loading control. PCR products were analyzed by electrophoresis through 1.2% agarose gels containing 0.1 mg/ml of ethidium bromide, and the gels were photographed using a UVP BioDocit system.

### Cloning of the human Brf1 promoter

Gene2Promoter analysis software program of Genomatix Suite  was utilized to identify a putative promoter for human Brf1. Putative transcription factor sites within the human Brf1 promoter were identified using MatInspector [53] of the Genomatix Suite. PCR primers flanking the Brf1 promoter sequence were designed with KpnI and BglII restriction sites and used to PCR amplify the promoter sequence from human genomic DNA and cloned into the KpnI and BglII sites of the promoterless pGL3 Basic vector (Promega).

### Luciferase Assays

Increasing concentrations of Brf1-pGL3 and Brf2-pGL3 [29] were transiently transfected using TransIT-LT1 (Mirus), as per the manufacturer's protocol in HeLa, MCF-7 and DU145 cells. pGL3-VAI and pGL3-U6 luciferase assays were performed as previously described [26]. In brief, the human U6 and VAI promoters were cloned into the promoterless pGL3 vector, generating pGL3-VAI and pGL3-U6. The dual-luciferase reporter assay system (Promega) was used to monitor luciferase activity in HeLa, DU 145 and MCF-7 cells as per the manufacturer's recommendations, using a Sirius single tube luminometer (Berthold). A Renilla luciferase vector (Promega) was co-transfected in all transfections to monitor transfection efficiency. Luciferase experiments were performed in triplicate or quadruplicate, repeated three independent times, and the data presented are representative experiments. All luciferase results are reported as relative light units (RLU): the average of the Photinus pyralis firefly activity observed divided by the average of the activity recorded from Renilla luciferase vector and graphed using GraphpadPrism3.03 (San Diego California USA).

### Western Blots

HeLa, MCF-7 and DU145 cells were harvested and nuclear extract was prepared as previously described [7]. Nuclear extract (25 μg) was separated on 10% SDS-PAGE gels, semi dry transferred to nitrocellulose, blocked 1 hour room temperature using nonfat milk in Phosphate Buffered Saline (PBS), pH 7.5. The blot(s) were incubated in primary antibodies: anti-actin (Santa Cruz), anti-Brf1 (CS407) or anti-Brf2 (CS1228) polyclonal antibodies, overnight at 4 degrees. The blot(s) were washed in 1× PBS and then incubated in secondary antibody at room temperature, then developed using ECF (for anti-rabbit-AP) or TMB substrate (Promega) (for anti-goat-HRP, Amersham) and photodocumented using a BioDocit system (UVP).

## Authors' contributions

SC was responsible for RT-PCR analysis, western blot and manuscript preparation. JJ cloned the Brf1 promoter and sequence alignments. IV conducted RNA pol III luciferase assays. LS conceived and coordinated study, performed Brf1 and Brf2 promoter luciferase assays, and drafted manuscript. All authors have read and approved the final manuscript.
